# Phylogenomic insights into the first multicellular streptophyte

**DOI:** 10.1016/j.cub.2023.12.070

**Published:** 2024-02-05

**Authors:** Maaike J. Bierenbroodspot, Tatyana Darienko, Sophie de Vries, Janine M.R. Fürst-Jansen, Henrik Buschmann, Thomas Pröschold, Iker Irisarri, Jan de Vries

**Affiliations:** 1 University of Goettingen, Institute for Microbiology and Genetics, Department of Applied Bioinformatics, Goldschmidtstr. 1, 37077 Goettingen, Germany; 2 University of Goettingen, Campus Institute Data Science (CIDAS), Goldschmidstr. 1, 37077 Goettingen, Germany; 3 University of Applied Sciences Mittweida, Faculty of Applied Computer Sciences and Biosciences, Section Biotechnology and Chemistry, Molecular Biotechnology, Technikumplatz 17, 09648 Mittweida, Germany; 4 University of Innsbruck, Research Department for Limnology, 5310 Mondsee, Austria; 5 Section Phylogenomics, Centre for Molecular Biodiversity Research, Leibniz Institute for the Analysis of Biodiversity Change (LIB), Museum of Nature, Hamburg, Martin-Luther-King Platz 3, 20146 Hamburg, Germany; 6 University of Goettingen, Goettingen Center for Molecular Biosciences (GZMB), Department of Applied Bioinformatics, Goldschmidtstr. 1, 37077 Goettingen, Germany

**Keywords:** Charophyta, streptophyte algae, multicellularity, phylogenomics, plant terrestrialization, plant evolution, ancestral character state

## Abstract

Streptophytes are best known as the clade containing the teeming diversity of embryophytes (land plants).^1^^,^^2^^,^^3^^,^^4^ Next to embryophytes are however a range of freshwater and terrestrial algae that bear important information on the emergence of key traits of land plants. Among these, the Klebsormidiophyceae stand out. Thriving in diverse environments—from mundane (ubiquitous occurrence on tree barks and rocks) to extreme (from the Atacama Desert to the Antarctic)—Klebsormidiophyceae can exhibit filamentous body plans and display remarkable resilience as colonizers of terrestrial habitats.^5^^,^^6^ Currently, the lack of a robust phylogenetic framework for the Klebsormidiophyceae hampers our understanding of the evolutionary history of these key traits. Here, we conducted a phylogenomic analysis utilizing advanced models that can counteract systematic biases. We sequenced 24 new transcriptomes of Klebsormidiophyceae and combined them with 14 previously published genomic and transcriptomic datasets. Using an analysis built on 845 loci and sophisticated mixture models, we establish a phylogenomic framework, dividing the six distinct genera of Klebsormidiophyceae in a novel three-order system, with a deep divergence more than 830 million years ago. Our reconstructions of ancestral states suggest (1) an evolutionary history of multiple transitions between terrestrial-aquatic habitats, with stem Klebsormidiales having conquered land earlier than embryophytes, and (2) that the body plan of the last common ancestor of Klebsormidiophyceae was multicellular, with a high probability that it was filamentous whereas the sarcinoids and unicells in Klebsormidiophyceae are likely derived states. We provide evidence that the first multicellular streptophytes likely lived about a billion years ago.

## Highlights

• Comprehensive phylogenomic analyses for 38 taxonomically diverse Klebsormidiophyceae
• Three-order system for the Klebsormidiophyceae, resilient land colonizers
• Deep divergence, 830 million years ago—long before embryophytes emerged
• Evidence that multicellularity emerged in streptophytes about a billion years ago

## Graphical abstract

**Figure:**
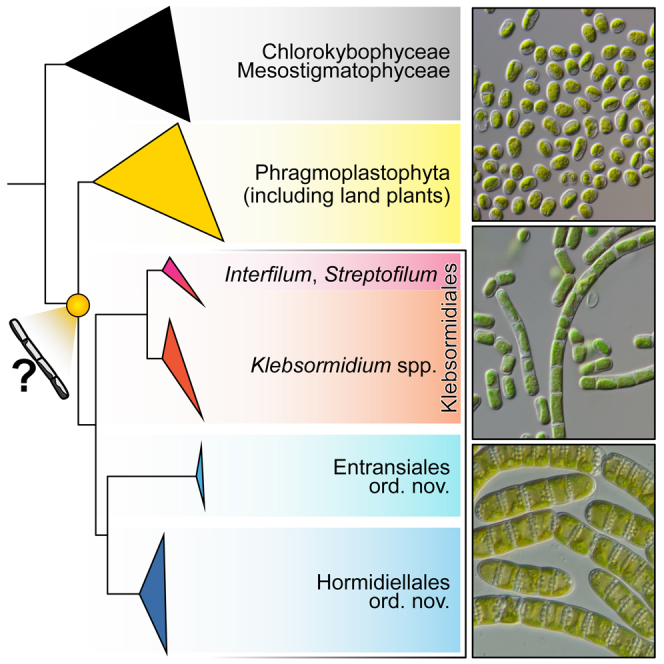

## Teaser

Bierenbroodspot et al. use comprehensive phylogenomic analyses to propose a three-order system for Klebsormidiophyceae, streptophyte algae that colonize terrestrial habitats but are distantly related to land plants. They trace the occurrence of multicellular growth that, based on molecular dating, might have emerged about a billion years ago.

## Results and discussion

### Klebsormidiophyceae are cosmopolitan colonizers of diverse habitats

**Figure 1:**
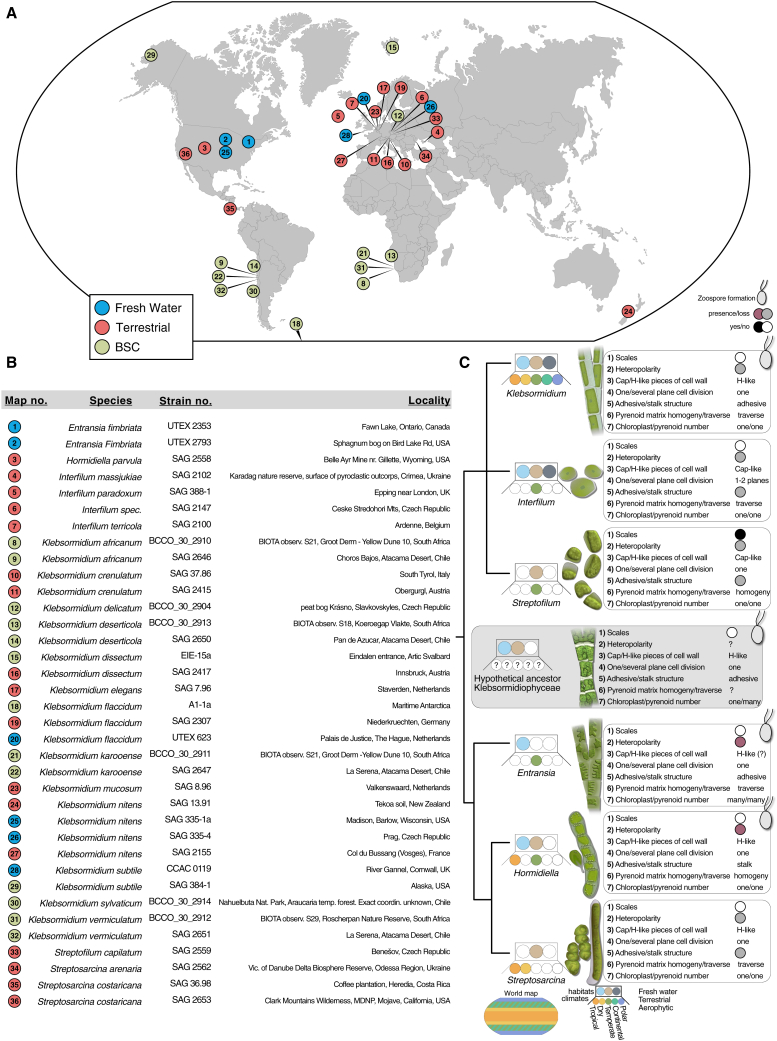
Biogeography of Klebsormidiophyceae (A) World map with all the klebsormidiophycean strains used within this study. An interactive map can be accessed under https://tinyurl.com/yph2s4ma. (B) Details on the strains of Klebsormidiophyceae used in this study. (C) Cladogram of the genera in Klebsormidiophyceae. Dots label their distribution across climate zones, habitats, and body plan diversity. Character information was guided by Mikhailyuk et al.^22^

In our sampling, we accounted for the class of Klebsormidiophyceae standing out among green algae by their resilience^6^^,^^7^ and habitat range. We obtained representatives that can be found in streams, rivers,^8^^,^^9^ lakeshores,^10^ bogs,^9^ soil,^11^ natural rocks in flat and mountainous regions,^12^ tree bark,^13^ acidic post-mining sites, freshwater bodies,^14^^,^^15^^,^^16^ sand dunes,^17^ biotic crusts of hot deserts,^18^ and human-shaped habitats such as urban walls^19^ and building façades.^20^^,^^21^ We sampled from the hottest (Atacama Desert) to the coldest (Antarctic) arid regions, from growing in freshwater to land, including representatives involved in forming biological soil crusts (BSCs). Overall, our sampling presents a worldwide distribution map for the ancient lineage of Klebsormidiophyceae including reference strains of described species, showcasing their habitat utilization and adaptability, ecological significance, and hidden diversity (Figures 1A and 1B).

The ability of Klebsormidiophyceae to dwell in diverse habitats is underpinned by a set of molecular physiological traits, such as those contributing to desiccation resistance. For example, *Klebsormidium crenulatum* can undergo regulated cell shrinkage under desiccation stress supported by the flexibility of its cell wall based on high levels of callose,^23^ special transglycanases,^24^ and the accompanying alterations in the actin cytoskeleton.^6^^,^^25^ Furthermore, in terrestrial habitats, temperature fluctuations are more pronounced compared to in aquatic environments. As a result, membrane fluidity becomes crucial, as it directly impacts the expression of genes involved in cold or heat responses.^26^^,^^27^^,^^28^ Owing to the broad distribution of habitat adaptations (Figure 1C), the emergence of the enabling traits needs to be projected onto a phylogenetic framework.

The class Klebsormidiophyceae comprises a minimum of five different genera: *Klebsormidium*, *Interfilum*, *Entransia*, *Hormidiella*, and *Streptosarcina*.^22^^,^^29^ Within *Interfilum* and *Klebsormidium*, seven main clades (A, B, C, D, E, F, and G) have been identified through the analysis of ITS and *rbc*L markers.^21^^,^^30^^,^^31^ To scrutinize the distribution and diversity of Klebsormidiophyceae, we studied 24 strains and included 14 previously published isolates in this dataset; we gave particular attention to sample densely within clade G, which is rich in species but currently scarce in sequence data. Preliminary phylogenetic analyses were performed using a representative dataset of 31 streptophyte taxa, employing two commonly used markers (*rbc*L, SSU; Figure S1). Owing to the incongruence between the phylogenetic trees built on individual markers, no robust reconstruction of the relationships within Klebsormidiophyceae was possible (Figure S1). Indeed, in line with previous studies, two major problems were unsolved. The first is the lack of monophyly of *Klebsormidium*. The type species of *Klebsormidium* (*K. flaccidum*; clades B/C) represents the sister to *Interfilum* based on *rbc*L and ITS sequences, whereas the rest of *Klebsormidium* (clades D–G) remains separated from those. If true, this has crucial taxonomical consequences because of the priority rule: the genus *Interfilum* is older than *Klebsormidium* and would have priority if these phylogenetic results were robust. The second unresolved problem was the phylogenetic position of the genus *Streptofilum* within the Streptophyta. According to the phylogenetic analyses of the combined *rbc*L-SSU and 44 chloroplast genes, *Streptofilum* is suggested to represent a separate lineage outside of Klebsormidiophyceae^22^^,^^32^; however, the phylogeny remains unresolved. Overall, the resolution of these markers is not powerful enough to resolve the complex evolutionary history of the Klebsormidiophyceae*.* To scrutinize the internal genetic structure of Klebsormidiophyceae, we took advantage of the higher resolving power of phylogenomics based on hundreds of nuclear genes.

### A phylogenomic framework and three-order system for Klebsormidiophyceae

**Figure 2:**
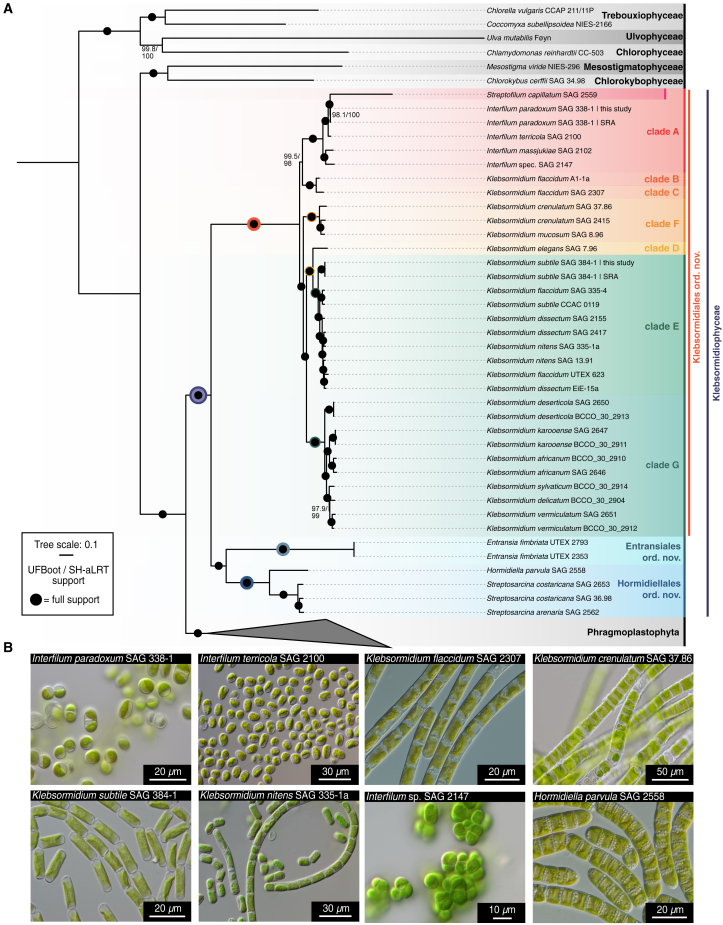
A new three-order system of Klebsormidiophyceae based on phylogenomics (A) Maximum likelihood phylogenetic analyses based on 845 loci and the complex LG+C60 mixture model. UFBoot2 and an SH-like aLRT branch support test were employed. A dot labels full branch support; a colored halo around the dots labels the support indices for major clades. For additional assessment of robustness (gene jackknife and topology tests), see Figures S1B and S1C; a time-calibrated tree is shown in Figure S2. (B) A selection of the morphological diversity found across Klebsormidiophyceae. Scale bars are labeled in each of the micrographs.

Using the Illumina NovaSeq6000 platform, we sequenced the transcriptomes of 24 isolates of Klebsormidiophyceae, including 15 strains of *Klebsormidium*, 4 *Interfilum*, and one of each *Hormidiella* and *Streptofilum*, as well as three isolates of *Streptosarcina* collected from five continents and various habitats from all climate zones. In total, we sequenced 1.407 billion paired-end transcriptomic reads, providing more than 423 gigabases of raw sequence information. To complement our dataset, we integrated these data with 14 previously published transcriptomes and 24 additional samples of algae and land plants (see STAR Methods). With a focus on 845 densely sampled loci, we used maximum likelihood with the complex LG+C60 mixture model to construct a robust phylogenomic tree (Figure 2).

The maximum likelihood tree was fully resolved and accurately represented the accepted phylogeny for the major groups of the green lineage (Chloroplastida). The tree confirmed the positioning of the Klebsormidiophyceae as the sister group to Phragmoplastophyta^1^^,^^33^ with full support from various metrics (Figure 2). Within Klebsormidiophyceae, two major clades emerged: (1) a clade consisting of *Entransia*, *Hormidiella*, and a monophylum of the three *Streptosarcina* strains that branched sister to (2) the clade of the other Klebsormidiophyceae, consisting of clades A to G, formed by the genera *Interfilum*, *Klebsormidium*, and *Streptofilum*. Importantly, we recovered a large monophyletic clade of *Klebsormidium* spp. (23 strains, full support, clades D to G) and a monophyletic clade formed by the genera *Interfilum* and *Streptofilum* (5 strains, full support; clade A), and *Klebsormidium flaccidum* (A1-1a) and *Klebsormidium flaccidum* (SAG 2307); the latter two formed a monophylum sister to the *Interfilum*/*Streptofilum* clade, representing clades B and C.^21^ Thus, while the rest of the genus *Klebsormidium* was monophyletic, some species therein were recovered as paraphyletic and will require focused investigations.

In addition to commonly used branch support metrics, we calculated gene jackknife proportions^34^^,^^35^ (Figure S1B) to further assess the robustness of our phylogenetic hypothesis. With relatively short, concatenated alignments (10,000 aligned amino acids), 57.6% of the branches were supported by >95% gene jackknife proportions, which increased to 78.0% (30,000) and 89.8% (60,000) with about one-third and two-thirds of the total data, respectively. With the longest gene jackknife replicates (85,000) representing almost the entirety of the concatenated alignment, 94.9% and 96.6% of the branches were recovered 100% and >95% of the time, respectively. Only two branches received low gene jackknife support with the longest pseudo-replicates: the sister group relationships of *Interfilum* and *Klebsormidium* clade B/C and the position of *Isoetes* in the outgroup. Despite the high support for the position of *Streptofilum* (it received 100% gene jackknife proportions with 60,000-long pseudo-replicates), its long branch stands out. Thus, the relationships of clades A, B, and C will require additional phylogenetic treatment and potentially taxonomic revision. To further assess the robustness of our gross topology and three-order system, we tested the support of our data for alternative relationships of the three proposed orders and *Streptofilum*, all of which were rejected with very low approximately unbiased (AU) p values (Figure S1C).

Our phylogenomic analyses recover a topology that features a deep split in the Klebsormidiophyceae. According to our molecular clock analyses, this split happened 830.83 (589.39 to 1074.43 95% HPD age estimates) million years ago (Figure S2A). The resulting clade again split 666.46 (407.49 to 896.32 95% HPD age estimates; *Entransia*–*Hormidiella*/*Streptosarcina*) million years ago and 444.63 (264.3 to 563.56 95% HPD age estimates; deepest bifurcation within Klebsormidiales) million years ago. We thus recover major diversifications in Klebsormidiophyceae that are deeper than the deepest divergence in Embryophyta (for timing on embryophytes, see Morris et al.^36^; overall, our molecular clock data agree with current estimates on the timing of early streptophyte evolution^37^). We therefore propose to account for this deep genetic structure by dividing Klebsormidiophyceae into a three-order system (Table 1): Klebsormidiales ord. nov. with the genera *Interfilum*, *Streptofilum*, and *Klebsormidium* (forming a fully supported monophylum that encompasses the clades and grades A to G), Hormidiellales ord. nov., and Entransiales ord. nov. (Figure 2).

**Table 1:** Revision of the class Klebsormidiophyceae and its orders

Taxonomical and nomenclatural problems of the class Klebsormidiophyceae and justification for emendation. Jeffrey^38^ was the first who used the name Klebsormidiophyceae without formal description of the class. Later on, van den Hoek and co-authors^39^ separated this group based on ultrastructural features of flagellar apparatus, presence of VII and VIII types of mitosis and cytokinesis. The authors proposed two orders, Klebsormidiales and Coleochaetales, within Klebsormidiophyceae. Unfortunately, the class Klebsormidiophyceae was not formally described. The Latin diagnosis was not delivered, and type order was not defined. In the later publications as justification for the establishment of the class, Stewart and Mattox^40^ was cited. However, the authors also did not formally describe the order and refer to the Latin diagnosis of Silva et al.,^41^ who corrected the generic name *Klebsormidium* within the family but did not formally describe the family Klebsormidiaceae or order Klebsormidiales (see Silva^42^). As results of our findings, we formally emend the class Klebosormidiophyceae according to the International Code of Nomenclature for algae, fungi, and plants (ICN) and proposed three orders as follows:
**Class Klebsormidiophyceae C. Jeffrey ex Guiry 2023, Notulae Algarum 303: 1; emend.** Description: Cell division by the formation of cleavage furrow (type VII according to van den Hook et al.^39^). Flagellar apparatus associated with MLS. Cell wall bipartite or H-like, cap-like, or with scales (?). Zoospores (if present) unilateral possess two flagella covered with squared scales and hairs and inserted asymmetrically, without stigma and cell wall-less. Comprises filaments, packages or unicells with parietal chloroplast. Type order (designated here): Klebsormidiales ordo nov. **Order Klebsormidiales ordo nov.** Description: With features of the class. Diagnosis: Differ by the absence of heteropolarity in comparison to Entransiales and Hormidiellales (see below). Type family (designated here): Klebsormidiaceae fam. nov. **Family Klebsormidiaceae fam. nov.** Description: With features of the order. Type genus (designated here): *Klebsormidium* Silva, Mattox & Blackwell 1972, Taxon 21: 643. Note: Currently, it contains three genera, *Klebsormidium, Interfilum* Chodat,^43^ and Streptofilum Mikhailyuk & Lukešová 2018 (Mikhailyuk et al. 2018).
**Order Entransiales ordo nov.** Description: comprises unbranched filaments with H-like cell wall, cells with parietal chloroplast containing several pyrenoids. Asexual reproduction via zoospores (if present). Zoospore germination often with formation of amorphous adhesive holdfast and tendency to heteropolarity (forming tapering spines at the tip of germinating filaments). Currently contains only one genus (*Entransia*). Diagnosis: Differ by the presence of heteropolarity in comparison to Klebsormidiales (see above). Type family (designated here): Entransiaceae fam. nov. **Family Entransiaceae fam. nov.** Diagnosis: with characteristic of order Entransiales. Type genus (designated here): *Entransia* E.O. Hughes 1948, Amer. J. Bot. 35: 427. Note: Currently it contains only two species: *E. fimbriata* and a doubtful *E. dichloroplastes* Prescott.^44^
**Order Hormidiellales ordo nov.** Description: comprises branched or unbranched filaments and packets with H-like cell wall, cells with parietal chloroplast containing one pyrenoid. Asexual reproduction via unilateral zoospores without stigma. Zoospore germination often with formation of stalk in *Hormidiella* and tendency to heteropolarity or without adhesive structure (*Streptosarcina*). Diagnosis: Differ genetically in comparison to the other orders. Type family (designated here): Hormidiellaceae fam. nov. **Family Hormidiellaceae fam. nov.** Diagnosis: with characteristic of order Hormidiellaceae. Type genus (designated here): *Hormidiella* Iyengar & Kanthamma 1940, J. Indian Bot. Soc. 19: 165. Note: Currently, it contains two genera *Hormidiella* and *Streptosarcina* Mikhailyuk & Lukešová 2018 (Mikhailyuk et al. 2018).

The enigmatic *Entransia* was first described from Nova Scotia^45^ and for a long time tentatively placed in the Zygnematophyceae. We here describe it as Entransiales ord. nov., consisting of a fully supported clade and the family Entransiaceae fam. nov.; Entransiales ord. nov. form a clade with the Hormidiellales ord. nov. Previous molecular phylogenetic analyses based on several genes demonstrated that *Entransia* is a member of Klebsormidiophyceae.^46^^,^^47^^,^^48^ Cook^49^ conducted a detailed study of the two available isolates of *Entransia* and firmly anchored its position within Klebsormidiophyceae solely based on morphological and cytological features. The morphological characters shared by *Entransia* and other Klebsormidiophyceae include cylindrical cells, unbranched filaments, parietal laminated chloroplast, H-shaped cross-wall pieces, and asexual reproduction by fragmentation as well as zoospores and aplanospores.

*Hormidiella* is here described as part of Hormidiellales ord. nov. and the family Hormidiellaceae fam. nov. Both *Entransia* and *Hormidiella* have a centriole pair proximal to the nucleus,^50^ the tendency for upright growth, and differentiation into three types of vegetative cells: cells with adhesive structures (*Entransia*) or stalks (*Hormidiella*), normal vegetative cells, and upper tapered cells. Both genera also produce asexual zoospores. The sister genus *Streptosarcina* appears to have lost asexual zoospores during the adaptation to the arid habitats and developed instead 2D division,^22^ which may serve as protection from desiccation; the mechanisms and induction of branching in *Streptosarcina*, however, remain obscure. Some traits are also shared by G-clade *Klebsormidium* spp. and *Hormidiella*. Morphological investigation of mature cultures demonstrated that these organisms possess relative coin-like cells and filaments disintegrating into short filaments^22^ with tapered end cells. Representatives of the G clade have generally smaller cell sizes that could also be adaptative to desiccation.

*Streptofilum* is here recovered as nested in the clade of *Interfilum* (the Klebsormidiales). In the description of *Streptofilum*, the authors^22^ noted a shared feature with mature vegetative cells of *Mesostigma*: a scaly cell wall. Other representatives of the Streptophyta, including some mosses and ferns, possess cell-covering scales only in asexual reproductive stages (zoospores). Hence, while *Streptofilum* and *Interfilum* might have superficial similarities in morphology and ecology, the ultrastructure of the vegetative cells showed organic scales in *Streptofilum* (in contrast to *Interfilum*). It is important to note that Pierangelini et al.^51^ explored the ecophysiological performance of *Streptofilum*, *Streptosarcina*, and *Hormidiella*, finding that common photosynthetic adaptations occur in similar habitats, driven by light and dehydration, irrespective of phylogenetic relationships within Klebsormidiophyceae.

Overall, while there is a set of shared and distinct traits to all orders in Klebsormidiophyceae, our phylogenomic data establish a robust and unequivocal backbone of Klebsormidiophyceae evolution consisting of two deep dichotomies nested within an even deeper dichotomy in Klebsormidiophyceae—a split more distant in the past than the split of all land plants. We next used this new phylogenomic framework and three-order system to understand the evolutionary history of key traits.

### The first multicellular streptophyte emerged around one billion years ago

Land plants are among those photosynthetic eukaryotes with the most complex true multicellularity. The evolutionary emergence of streptophyte multicellularity is thus one of the general interest topics. Here, streptophyte algae hold important information and surprises. Among those streptophyte algae most closely related to land plants, the Zygnematophyceae, we find unicells and (at times branched) filaments. This stands in stark contrast to other (phragmoplastophytic) streptophyte algae, which have parenchymatous growth (Coleochaetophyceae) or even erect growth with organs and thus 3D growth^52^ (Charophyceae). It was inferred that the common ancestor of Zygnematophyceae likely underwent reduction and might have even ancestrally transitioned to a unicellular body.^53^ To understand the propensity for multicellularity among Phragmoplastophyta, one must turn to its sister group: the Klebsormidiophyceae.

**Figure 3:**
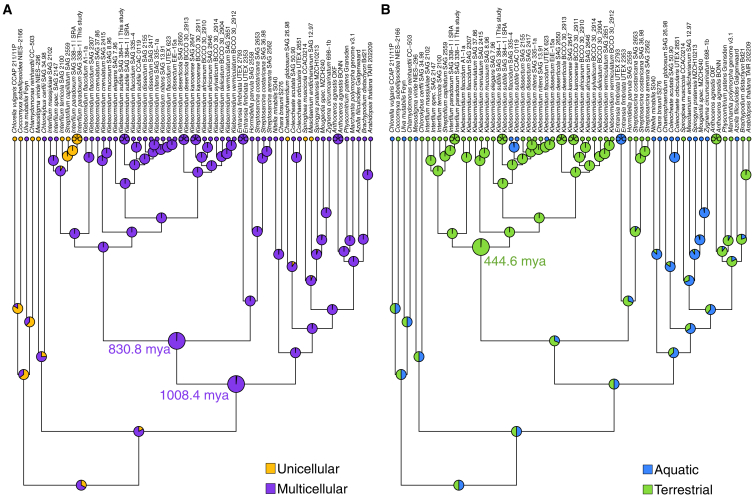
Ancestral character state reconstruction of body plan and habitat characters across more than 800 million years of klebsormidiophycean evolution (A) To examine the ancestral character states of growth types in unicellular or multicellular organisms, coding schemes represented varying levels of complexity and hypotheses regarding the homology of growth types. The shown color-coded character state distributions represent yellow for unicellular growth and purple for multicellular growth *sensu lato* (including sarcinoid, filamentous, and parenchymatous growth). Note ancestor nodes for (1) Klebsormidiophyceae and Phragmoplastophyta 1008.4 million years ago (mya) and (2) Klebsormidiophyceae 830.8 mya. (B) To examine the ancestral habitats of the Klebsormidiophyceae, we coded habitat occurrences of the species as blue for aquatic and green for terrestrial. Note the terrestrial ancestor of Klebsormidiales, which lived 444.6 mya. Divergence dating is based on the molecular clock analyses shown in Figure S2. All ancestral character state reconstructions were done with a symmetric rates model because we consider most conservative by making the least assumptions about exchange rates; different coding schemes and asymmetric models are shown, alongside a key gene family salient to terrestrial adaptation (PAL), in Data S1.

Klebsormidiophyceae includes sarcinoid (a thallus comprised of cellular colonies organized in a three-dimensional, packet-like structure), uniserial unbranched filaments, and filaments that easily disintegrate into unicells.^22^^,^^31^ According to phylogenetic reconstruction based on single (or few) gene(s) or multigene approaches,^21^^,^^22^^,^^29^ the position of the sarcinoid morphotype is spread among different clades and probably is derived from a filamentous type. Interestingly, both sarcinoid and filamentous morphotypes can be present within the same species or genus, for example in *Streptosarcina costaricana* or *Interfilum*.^22^^,^^29^ This could represent an advantage for colonizing different terrestrial substrates, due to the lower surface-to-volume relationship of large cell assemblages or the possibility of crust formation by filaments. To understand the evolution of growth types in Klebsormidiophyceae and streptophytes in general, we conducted ancestral character state reconstructions (ACSRs) by maximum likelihood. Multiple data coding strategies were employed, particularly focused on the type of cellular growth (Figure 3; Data S1). In the simplest coding pattern, we recovered full support for a multicellular ancestor of Klebsormidiophyceae (posterior probability [PP] of 0.995) and for a multicellular ancestor of Klebsormidiophyceae and Phragmoplastophyta (PP of 0.984). If we employ a three-character coding, distinguishing between unicells, sarcinoid cell packages, and filamentous or more complex body plans, we also recover a multicellular ancestor of Klebsormidiophyceae and Phragmoplastophyta, with a likely filamentous (PP of 0.843 and 0.906) and less likely sarcinoid (PP of 0.152 and 0.092) body plan (Data S1). Thus, the first multicellular streptophyte likely lived around a billion years ago (1008.45 mya; 777.94 to 1263.04 95% HPD age estimates; Figures 3 and S2 and Data S1).

What does this mean for the ability to grow on land? While some streptophyte lineages, such as *Mesostigma* or *Chlorokybus*, are rare algae with very narrow ecological niches, one of the most striking successes in colonizing terrestrial habitats can be found in *Klebsormidium*. *Klebsormidium* spp. are one of the few eukaryotes that are capable of forming BSCs on their own or together with cyanobacteria, mosses, or lichens.^54^ We coded habitat occurrence and, using our newly established phylogenetic backbone, performed ACSR to identify the history of habitat shifts in Klebsormidiophyceae. No clear pattern support was recovered for the deep ancestors, but a full support (PP of 0.994) for ancestrally terrestrial Klebsormidiales (Figure 3B, node with 444.63 my [264.3 to 563.56 95% HPD] age estimates). This has implications for the evolution of physiological traits. *Interfilum* and *Klebsormidium* as well as *Hormidiella* and *Streptosarcina* are known to produce similar special mycosporine-like amino acids acting as UV-protectors^55^—whereas *Streptofilium* had MAAs with different peaks and retention times.^51^ Yet, another source of protectants could be phenylpropanoid-derived compounds. The first enzyme in the pathway, phenylalanine-ammonia lyase (PAL), was thought to be a land plant innovation that was proposed to have emerged via horizontal gene transfer from soil-associated bacteria.^56^ However, when the genome of *Klebsormidium nitens* was published,^57^ it was found to have a PAL homolog,^58^^,^^59^ raising the question of when the streptophyte PAL emerged. In our dataset, we found candidates for PAL for other Klebsormidiales (*Klebsormidium* and *Interfilum*), which formed a fully supported clade with bacterial PALs (Data S1). No homologs outside of Klebsormidiales—neither other Klebsormidiophyceae nor other algae—were found. Overall, this suggests that the ancestor of *Klebsormidium* and *Interfilum* may have acquired a bacterial PAL from soil-associated bacteria independent from the origin in land plants.^56^

Our data suggest that the ability for filamentous growth is ancient in streptophytes—it emerged at least one billion years ago. Recently, Hess et al.^53^ found that the Zygnematophyceae (the algal sister lineage of land plants) might have (re-)gained a filamentous body plan multiple times independently—aligning with the shared co-expression of cell division regulators.^60^^,^^61^ Our ACSR on Klebsormidiophyceae might help explain this: the molecular machinery for filamentous growth might be a set of homologous genes—including, e.g., RHO of plant (ROP GTPase)^62^—shared since the last common ancestor of Klebsormidiophyceae and Phragmoplastophyta. Hence, there is an ancient one-billion-year-old genetic potential for multicellularity among streptophytes, explaining the propensity to become multicellular. This propensity, building on shared genetic potential, was realized multiple times throughout streptophyte evolution, sometimes resulting in very complex multicellular organisms like *Chara* and land plants, other times in manifesting in mere filamentous growth. That there is a smooth transition between unicellular and multicellular growth is palpable when taking a closer look at, for example, *Interfilum* sp. SAG 2147, whose cells are often organized in clumps (Figure S2C). However, these clumps easily fall apart, resulting mainly in groups of two or four cells. Groups with four cells can be arranged longitudinally, suggesting that cytokinesis was solely transverse. However, in packs of four, it appears that the cell division plane has changed by 90° since the previous division. This indicates rotations in the cell division plane. Additionally, cell wall remodeling after cell division seems to be extensive and fast.

Filamentous Klebsormidiophyceae exhibit, like filamentous Zygnematophyceae, one of the simplest forms of multicellularity: non-branching (“1D”) filaments.^52^^,^^63^ A classical vegetative centripetal cleavage gives rise to these filaments.^64^^,^^65^ That said, some intricate molecular mechanisms known from land plants might be at play, foremost the ancient phytohormone auxin.^66^ Ohtaka et al.^67^ found that *Klebsormidium nitens* NIES-2285 alters its cell division and cell elongation upon auxin treatment, and it was later confirmed that *K. flaccidum* has a functional auxin efflux carrier^68^—which in land plants is key for polar auxin transport-mediated morphogenesis.^69^ Thus, some key morphogenic processes likely had a deep evolutionary origin in the first filamentous streptophyte.

The frequent loss and gain of filamentous growth suggest that this habit involves several independent genes, each with additional functions. Indeed, also unicellular green algae have most of the genes needed for multicellularity.^70^^,^^71^ This implies that the complete loss of these genes, even when the lineage reverts to a simpler growth type (likely unicells), is unlikely. This enables both forward and backward evolutionary transitions in body plans across many clades and millennia, as only one or a few genes need to undergo slight changes in their activity.

### Conclusion

Significant efforts have been made in the past decade to understand the phylogenetic relationships within streptophytes, particularly in relation to embryophytes, and the deep evolutionary origin of land plant traits.^33^^,^^53^^,^^57^^,^^61^^,^^72^^,^^73^^,^^74^^,^^75^^,^^76^^,^^77^ However, the evolutionary history of one of the defining traits of land plants remains debated: multicellularity and complex body plans. We investigated Klebsormidiophyceae using a phylotranscriptomic approach building on isolates from around the world to establish the deep genetic structure within Klebsormidiophyceae and their relation to Phragmoplastophyta. Through ACSR, we demonstrate that the common ancestor of Phragmoplastophyta and Klebsormidiophyceae was already a multicellular alga; this alga lived almost a billion years ago.

## STAR★Methods

### Key resources table

**Table:** 

REAGENT or RESOURCE	SOURCE	IDENTIFIER
**Critical commercial assays**		

DNAse I	Thermo Fisher, Waltham, MA, USA	N/A
Sigma’s Spectrum ™ Plant Total RNA Kit	Sigma, Saint Louis, MO, USA	N/A

**Deposited data**		

Assemblies, SuperTranscripts, BUSCO, Transdecoder, Decontamination, Orthofinder, PhyloPyPruner, Prequal, and concatenated Alignment	This study	https://doi.org/10.5281/zenodo.10058795
Alignment, improved with 845 loci	This study	https://doi.org/10.5281/zenodo.10405945
*Anthoceros agrestis* BONN	Li et al.^78^	https://www.hornworts.uzh.ch/static/jbrowse/?data=a_agr_bonn
*Anthoceros agrestis* OXF	Li et al.^78^	https://www.hornworts.uzh.ch/static/jbrowse/?data=a_agr_oxford
*Arabidopsis thaliana* genome TAIR V10	TAIR^79^	http://www.arabidopsis.org
*Azolla filiculoides* Galgenwaard genome	Li et al.^80^	https://www.fernbase.org
*Brachypodium distachyon* Bd21	The International Brachypodium Initiative^81^	https://phytozome-next.jgi.doe.gov/info/Bdistachyon_v3_1
*Chaetophaeridium globosum* SAG 26.98 transcriptome	Cooper and Delwiche^82^	https://figshare.com/articles/dataset/Green_algal_transcriptomes_for_phylogenetics_and_comparative_genomics/1604778
*Chara braunii* S276 genome	Nishiyama et al.^83^	https://bioinformatics.psb.ugent.be/orcae/overview/Chbra
*Chlamydomonas reinhardtii* CC-503 cw92 mt+	Merchant et al.^84^ Blaby et al.^85^	https://phytozome.jgi.doe.gov/pz/portal.html#!info?alias=Org_Creinhardtii
*Chlorella vulgaris* CCAP 211/11P	Cecchin et al.^86^	https://www.ncbi.nlm.nih.gov/Traces/wgs/wgsviewer.cgi?val=SIDB00000000
*Chlorokybus cerffii* SAG 34.98	de Vries et al.^72^ Irisarri et al.^87^	https://datadryad.org/stash/dataset/https://doi.org/10.5061/dryad.0gb5mkm25
*Coccomyxa subellipsoidea* NIES-2166	Blanc et al.^88^	https://www.ncbi.nlm.nih.gov/Traces/wgs/wgsviewer.cgi?val=AGSI00000000.1
*Coleochaete orbicularis* UTEX 2651	Ju et al.^89^	https://www.ncbi.nlm.nih.gov/sra/?term=SRR1594679
*Coleochaete scutata* SAG 50.90 transcriptome	de Vries et al.^72^	https://www.ncbi.nlm.nih.gov/Traces/wgs/wgsviewer.cgi?val=GFXZ00000000
*Entransia fimbriata* UTEX 2353	Herburger et al.^50^ Carpenter et al.^90^	https://www.ncbi.nlm.nih.gov/sra/?term=ERR364372
*Entransia fimbriata* UTEX 2793	Cooper and Delwiche^82^	https://www.ncbi.nlm.nih.gov/sra/SRX13042274
*Hormidiella parvula* SAG 2558	This study	https://www.ncbi.nlm.nih.gov/sra/SRR26030693
*Isoetes taiwanensis* Kuo4500	Wickell et al.^91^	https://genomevolution.org/coge/GenomeInfo.pl?gid=61511 and https://www.ncbi.nlm.nih.gov/bioproject/735564
*Interfilum massjukiae* SAG 2102	This study	https://www.ncbi.nlm.nih.gov/sra/SRR26030692
*Interfilum paradoxum* SAG 338-1, 1 kP code FPCO	Carpenter et al.^90^	http://www.onekp.com/public_data.html
*Interfilum paradoxum* SAG 338-1	This study	https://www.ncbi.nlm.nih.gov/sra/SRR26030661https://www.ncbi.nlm.nih.gov/sra/SRR26030670https://www.ncbi.nlm.nih.gov/sra/SRR26030681
*Interfilum spec.* SAG 2147	This study	https://www.ncbi.nlm.nih.gov/sra/SRR26030659
*Interfilum terricola* SAG 2100	This study	https://www.ncbi.nlm.nih.gov/sra/SRR26030656https://www.ncbi.nlm.nih.gov/sra/SRR26030657https://www.ncbi.nlm.nih.gov/sra/SRR26030658
*Klebsormidium africanum* BCCO_30_2910	This study	https://www.ncbi.nlm.nih.gov/sra/SRR26030691
*Klebsormidium africanum* SAG 2646	This study	https://www.ncbi.nlm.nih.gov/sra/SRR26030690
*Klebsormidium crenulatum* SAG 2415	Holzinger et al.^92^	https://www.ncbi.nlm.nih.gov/bioproject/PRJNA255200
*Klebsormidium crenulatum* SAG 37.86	This study	https://www.ncbi.nlm.nih.gov/sra/SRR26030688https://www.ncbi.nlm.nih.gov/sra/SRR26030689
*Klebsormidium delicatum* BCCO_30_2904	This study	https://www.ncbi.nlm.nih.gov/sra/SRR26030687
*Klebsormidium deserticola* BCCO_30_2913	This study	https://www.ncbi.nlm.nih.gov/sra/SRR26030686
*Klebsormidium deserticola* SAG 2650	This study	https://www.ncbi.nlm.nih.gov/sra/SRR26030685
*Klebsormidium dissectum* EiE-15a	Borchhardt et al.^93^ Rippin et al.^94^	https://www.ncbi.nlm.nih.gov/biosample/SRS3995479
*Klebsormidium dissectum* SAG 2155	Fajkus et al.^95^	https://www.ncbi.nlm.nih.gov/sra/?term=SRS8235162
*Klebsormidium dissectum* SAG 2417	This study	https://www.ncbi.nlm.nih.gov/sra/SRR26030684
*Klebsormidium elegans* SAG 7.96	This study	https://www.ncbi.nlm.nih.gov/sra/SRR26030682https://www.ncbi.nlm.nih.gov/sra/SRR26030683
*Klebsormidium flaccidum* A1-1a	Borchardt et al.^96^ Rippin et al.^94^	https://www.ncbi.nlm.nih.gov/biosample/?term=SRS3995480
*Klebsormidium flaccidum* SAG 2307	de Vries et al.^72^	https://www.ncbi.nlm.nih.gov/Traces/wgs/wgsviewer.cgi?val=GFXY00000000
*Klebsormidium flaccidum* SAG 335-4	Rindi et al.^21^ Fajkus et al.^95^	https://www.ncbi.nlm.nih.gov/sra/SRX10075817
*Klebsormidium flaccidum* UTEX 623	Nelson et al.^97^	https://www.ncbi.nlm.nih.gov/sra/SRS5605435
*Klebsormidium karooense* BCCO_30_2911	This study	https://www.ncbi.nlm.nih.gov/sra/SRR26030679https://www.ncbi.nlm.nih.gov/sra/SRR26030680
*Klebsormidium karooense* SAG 2647	This study	https://www.ncbi.nlm.nih.gov/sra/SRR26030678
*Klebsormidium mucosum* SAG 8.90	This study	https://www.ncbi.nlm.nih.gov/sra/SRR26030675https://www.ncbi.nlm.nih.gov/sra/SRR26030676https://www.ncbi.nlm.nih.gov/sra/SRR26030677
*Klebsormidium nitens* SAG 335-1a (UTEX 321, NIES-2285)	Rindi et al.^21^ Ju et al.^89^ Cooper and Delwiche^82^	https://www.ncbi.nlm.nih.gov/sra/SRX13042247, https://www.ncbi.nlm.nih.gov/sra/?term=SRS714566
*Klebsormidium nitens* SAG 13.91	Rindi et al.^21^ Fajkus et al.^95^	https://www.ncbi.nlm.nih.gov/sra/SRX10075818
*Klebsormidium subtile* CCAC 0119	Rindi et al., 2008 Carpenter et al.^90^	https://www.ncbi.nlm.nih.gov/sra/?term=ERS368238
*Klebsormidium subtile* SAG 384-1	Rindi et al., 2008 Fajkus et al.^95^	https://www.ncbi.nlm.nih.gov/sra/?term=SRS8235161
*Klebsormidium subtile* SAG 384-1	This study	https://www.ncbi.nlm.nih.gov/sra/SRR26030672https://www.ncbi.nlm.nih.gov/sra/SRR26030673https://www.ncbi.nlm.nih.gov/sra/SRR26030674
*Klebsormidium sylvaticum* BCCO_30_2914	This study	https://www.ncbi.nlm.nih.gov/sra/SRR26030671
*Klebsormidium vermiculatum* BCCO_30_2912	This study	https://www.ncbi.nlm.nih.gov/sra/SRR26030669
*Klebsormidium vermiculatum* SAG 2651	This study	https://www.ncbi.nlm.nih.gov/sra/SRR26030667https://www.ncbi.nlm.nih.gov/sra/SRR26030668
*Marchantia polymorpha* genome v3.1	Bowman et al.^98^	https://phytozome.jgi.doe.gov/pz/portal.html#!info?alias=Org_Mpolymorpha
*Mesostigma viride* CCAC 1140	Wang et al.^33^	https://db.cngb.org/search/assembly/CNA0002352/
*Mesotaenium endlicherianum* SAG 12.97	Cheng et al.^75^	https://www.ncbi.nlm.nih.gov/bioproject/PRJNA541331/
*Mougeotia* sp. MZCH 240	de Vries et al.^74^ Fürst-Jansen et al.^99^	https://www.ncbi.nlm.nih.gov/bioproject/PRJNA543475/
*Nitella mirabilis* S040	Ju et al.^89^	https://www.ncbi.nlm.nih.gov/Traces/wgs/wgsviewer.cgi?val=GBST01&search=GBST01000000&display=scaffolds
*Physcomitrium patens* Gransden	Lang et al.^100^	https://phytozome.jgi.doe.gov/pz/portal.html#!info?alias=Org_Ppatens
*Spirogloea muscicola* CCAC 0214	Cheng et al.^75^	https://www.ncbi.nlm.nih.gov/bioproject/PRJNA541068/
*Spirogyra pratensis* MZCH 10213	de Vries et al.^74^	https://www.ncbi.nlm.nih.gov/bioproject/PRJNA543475/
*Streptofilum capillatum* SAG 2559	This study	https://www.ncbi.nlm.nih.gov/sra/SRR26030666
*Streptosarcina arenaria* SAG 2562	This study	https://www.ncbi.nlm.nih.gov/sra/SRR26030665
*Streptosarcina costaricana* SAG 2653	This study	https://www.ncbi.nlm.nih.gov/sra/SRR26030664
*Streptosarcina costaricana* SAG 36.98	This study	https://www.ncbi.nlm.nih.gov/sra/SRR26030662https://www.ncbi.nlm.nih.gov/sra/SRR26030663
Treefile (main tree)	This study	https://doi.org/10.5281/zenodo.10406003
*Ulva mutabilis* Føyn	De Clerck et al.^101^	https://bioinformatics.psb.ugent.be/orcae/overview/Ulvmu
*Zygnema circumcarinatum* SAG 698-1b	Feng et al.^61^	https://phycocosm.jgi.doe.gov/Zygcir6981b_2/Zygcir6981b_2.home.html

**Experimental models: Organisms/strains**		

*Hormidiella parvula SAG 2558*	This study	N/A
*Interfilum massjukiae* SAG 2102	This study	N/A
*Interfilum paradoxum* SAG 338-1	This study	N/A
*Interfilum spec.* SAG 2147	This study	N/A
*Interfilum terricola* SAG 2100	This study	N/A
*Klebsormidium africanum* BCCO_30_2910	This study	N/A
*Klebsormidium africanum* SAG 2646	This study	N/A
*Klebsormidium crenulatum* SAG 37.86	This study	N/A
*Klebsormidium delicatum* BCCO_30_2904	This study	N/A
*Klebsormidium deserticola* BCCO_30_2913	This study	N/A
*Klebsormidium deserticola* SAG 2650	This study	N/A
*Klebsormidium dissectum* SAG 2417	This study	N/A
*Klebsormidium elegans* SAG 7.96	This study	N/A
*Klebsormidium karoense* BCCO_30_2911	This study	N/A
*Klebsormidium karoense* SAG 2647	This study	N/A
*Klebsormidium mucosum* SAG 8.90	This study	N/A
*Klebsormidium subtile* SAG 384-1	This study	N/A
*Klebsormidium sylvaticum* BCCO_30_2914	This study	N/A
*Klebsormidium vermiculatum* BCCO_30_2912	This study	N/A
*Klebsormidium vermiculatum* SAG 2651	This study	N/A
*Streptofilum capillatum* SAG 2559	This study	N/A
*Streptosarcina arenaria* SAG 2562	This study	N/A
*Streptosarcina costaricana* SAG 2653	This study	N/A
*Streptosarcina costaricana* SAG 36.98	This study	N/A

**Software and algorithms**		

ApplyPPPFormat (apply Phylopypruner format)	This study	https://github.com/mjbieren/Phylogenomics_klebsormidiophyceae
BUSCO v.5.0.0	Seppey et al.^102^	https://busco.ezlab.org
ClipKIT	Steenwyk et al.^103^	https://github.com/JLSteenwyk/ClipKIT
FASTQC	Babraham Institute	www.bioinformatics.babraham.ac.uk/projects/fastqc
FilterPPPResult	This Study	https://github.com/mjbieren/Phylogenomics_klebsormidiophyceae
GPDS (Get Positive DataSet)	This study	https://github.com/mjbieren/Phylogenomics_klebsormidiophyceae
IQ-Tree2 v2.2.2.7	Nguyen et al.^104^	http://www.iqtree.org
MAFFT v7.310	Katoh and Standley^105^	https://mafft.cbrc.jp/alignment/software/
ModelFinder	Kalyaanamoorthy et al.^106^	http://www.iqtree.org/ModelFinder/
OSG (Orthogroup Sequence Grabber) and COGS (Combine Orthogroup Sets)	This study	https://github.com/mjbieren/Phylogenomics_klebsormidiophyceae
Phytools	Revell^107^	https://cran.r-project.org/web/packages/phytools/index.html
Posterior Mean Site Frequency Profiles	Wang et al.^108^	Implemented in IQ-Tree http://www.iqtree.org
PREQUAL	Whelan et al.^109^	https://github.com/simonwhelan/prequal
Re-routing method according to Yang 1995	Yang^110^	N/A
Transcdecoder v.5.5.0	Brian J. Haas	https://github.com/TransDecoder/TransDecoder/releases
Trimal v1.4.rev15	Capella-Gutierrez et al.^111^	http://trimal.cgenomics.org
Trimmomatic v0.36	Bolger et al.^112^	http://www.usadellab.org/cms/?page=trimmomatic

### Resource availability

#### Lead contact

Further information and requests for resources and reagents should be directed to and will be fulfilled by the lead contact, Jan de Vries (devries.jan@uni-goettingen.de).

#### Materials availability

This study did not generate new unique reagents.

#### Data and code availability

• RNA-seq data have been deposited at the NCBI under the BioProject accession PRJNA1013714 and the Sequence Read Archive under the accessions SRR26030656-SRR26030693; all data are publicly available as of the date of publication. Accession numbers are additionally listed in the key resources table.
• RNA-seq FastQC reports, BUSCO scorings, transcriptome assemblies, supertranscripts,TransDecoder outputs, decontaminated fasta files, Orthofinder orthogroups, Phylopypruner result, PREQUAL, MAFFT ginsi, and clipkit results, and the preliminary concatenated alignment file and the tree files can be found on Zenodo https://doi.org/10.5281/zenodo.10058795 — the final alignment with all 845 loci can be found on Zenodo https://doi.org/10.5281/zenodo.10405945 and the final tree file under https://doi.org/10.5281/zenodo.10406003
• The source code for the novel tools discussed in the paper (See RESOURCE TABLE - Software and Algorithms) are available on the GitHub link https://github.com/mjbieren/Phylogenomics_klebsormidiophyceae — any additional computational analyses, not pertaining to these tools, were conducted using established software and are properly referenced in the methods section. Corresponding batch and/or python scripts are also found within the GitHub link.

### Experimental model and subject details

#### Algal strains

Strains were obtained from the Culture Collection of Algae at Göttingen University^113^ (SAG). Six authentic strains representing the recently described species of *Klebsormidium*^31^ were received from one of the authors (AL) and officially deposited in at the Culture collection at Institute of Soil Biology (BCCO), Ceske Budejovice, Czech Republic. All strains were cultivated in 3NBBM (medium 26a^114^) at 18°C under full-spectrum fluorescent lamps (25-35 μmol photons m^-2^ s^-1^; 14:10h light-dark cycle).

### Method details

#### Light microscopy

High-resolution images of the studied strains were done with Olympus BX-60 microscope (Olympus, Japan) with DIC equipped with a ProgRes C14plus camera and the ProgRes CapturePro Software (version 2.9.01) (JENOPTIK AG, Jena, Germany). All investigated strains were examined at the 21^st^ day of cultivation.

#### RNA isolation

For the RNA extraction of 24 different strains, 50 mL of 21-day old liquid culture were centrifuged for 5 min at 20°C and 11000 rpm and the supernatant was removed. The pellet was transferred into the Tenbroek tissue homogenizer and each sample was manually disrupted during 10 min on ice. RNA extraction was done using the Spectrum Plant Total RNA Kit (Sigma-Aldrich Chemie GmbH, Germany) according to the manufacturer’s instructions. DNAse I treatment (Thermo Fisher, Waltham, MA, USA) was applied to the RNA samples, and their quality and quantity were assessed using a 1% agarose gel with a SDS stain, and Nanodrop (Thermo Fisher), respectively. The RNA samples were shipped on dry ice to Novogene (Cambridge, UK).

#### RNAseq and transcriptome assembly

At Novogene (Cambridge, UK), the samples underwent quality checks using a Bionanalyzer (Agilent Technologies Inc., Santa Clara, CA, USA), and library preparation was performed based on polyA enrichment and using directional mRNA library preparation. The libraries were quality checked and sequenced using the NovaSeq 6000 platform (Illumina) with Novogene dual adapters: 5′- AGATCGGAAGAGCGTCGTGTAGGGAAAGAGTGTAGATCTCGGTGGTCGCCGTATCATT-3′ for read 1 and 5′- GATCGGAAGAGCACACGTCTGAACTCCAGTCACGGATGACTATCTCGTATGCCGTCTTCTGCTTG-3′

Additionally, we downloaded RNAseq data for 14 different Klebsormidiophyceae species, including *Entransia fimbriata* UTEX 2353 (ERS368240; Carpenter et al.^90^), *Entransia fimbriata* UTEX 2793 (SRR16849194), *Interfilum paradoxum* SAG 338-1 (ERS1830152; Carpenter et al.^90^), *Klebsormidium crenulatum* SAG 2415 (SRS693696, SRS693678, SRS693690; Holzinger et al.^92^), *Klebsormidium dissectum* EiE-15a (SRS3995479; Borchhardt et al.^93^; Rippin et al;^94^), *Klebsormidium flaccidum* A1-1a (SRS3995480; Borchhardt et al.^96^; Rippin et al;^94^), *Klebsormidium flaccidum* SAG 2307 (SRP115828, SRP116582; de Vries et al.^72^), *Klebsormidium flaccidum* SAG 335-4 (SRS8235163; Fajkus et al.^95^), *Klebsormidium flaccidum* UTEX 623 (SRS5605435; Nelson et al.^97^)*, Klebsormidium nitens* SAG 2155 (SRP305831; Fajkus et al.^95^), *Klebsormidium nitens* SAG 13.91 (SRS8235164; Fajkus et al.^95^), *Klebsormidium nitens* SAG 335-1a (SRS10979560, SRS714566; Ju et al.^89^; Cooper and Delwiche^82^), *Klebsormidium subtile* CCAC 0119 (ERS368238; Carpenter et al.^90^), and *Klebsormidium subtile* SAG 384-1 (SRS8235161; Fajkus et al.^95^). All samples' transcriptomes were assembled *de novo* using Trinity v2.11.0 (Haas et al.^115^) after adapter trimming with Trimmomatic^112^ (--trimmomatic "ILLUMINACLIP:novogene_adapter_sequences.fa:2:30:10:2:keepBothReads LEADING:3 TRAILING:3 MINLEN:36″). SuperTranscripts (Davidson et al.^116^) were inferred by collapsing splicing isoforms using the Trinity implementation. The completeness of the transcriptomes was assessed with BUSCO v5.4.3 (Seppey et al.^102^) using the 'eukaryota_odb10′ reference set. The BUSCO completeness of all newly assembled transcriptomes was on average of 90.62% (see data on Zenodo, https://doi.org/10.5281/zenodo.10058795). Protein-coding genes were identified using Transdecoder v5.5.0, with *Klebsormidium nitens*^57^ (NIES-2285) as the reference in BLASTP searches, retaining only the longest open reading frame per transcript (--single_best_only).

#### Dataset construction for phylotranscriptomics

To remove potential contaminants, we conducted sequence similarity searches against a comprehensive database that included proteins from various sources. These sources include *Klebsormidium nitens* (NIES-2285),^57^ as well as potential contaminants such as RefSeq^117^ representative bacterial genomes (11,318 genomes), fungi (2,397), all available viruses, archaea (1,833), and plastid genes (78,2087). We employed MMseqs2 (Steinegger and Söding^118^) for the search, using an iterative approach with increasing sensitivities and maintaining a maximum of 10 hits (--start-sens 1 --sens-steps 3 -s 7 --alignment-mode 3 --max-seqs 10). To ensure stringent decontamination, we retained, with the help of GPDS (See key resources table - Software and Algorithms), only sequences that showed the best match to predicted Klebsormidiophyceae nuclear proteins for phylogenetic analysis. For each type of contaminant (bacteria, fungi, viruses, archaea, and plastids), separate files were automatically generated, which can be accessed at Zenodo under https://doi.org/10.5281/zenodo.10058795.

#### Phylotranscriptomic analysis

To infer orthogroups, Orthofinder v2.5.4 (Emms and Kelly^119^) was employed using a species tree following the approach of Leebens-Mack et al.^2^ The species tree included representation from chlorophytes, *Chlorokybus cerffii* SAG 34.98, *Mesostigma viride* NIES-296, and various Phragmoplastophyta (see data deposited on Zenodo, https://doi.org/10.5281/zenodo.10058795). This tree also included all the Klebsormidiophyceae with unresolved relationships.

From a total of 1,761,660 orthogroups, 16,410 were selected through taxonomic group filtering with the help of OSG (See key resources table - Software and Algorithms). This selection criterion required the presence of at least one sequence from each of the 10 main different taxonomic groups out of the total 14. Additional information regarding the taxonomic group ordering can be found on Zenodo.

Homologous sets were aligned using MAFFT^105^ v7.304 with default settings, and maximum likelihood inference was performed using IQ-Tree2^104^^,^^120^ multicore version 2.2.2.7. The analysis involved fast searches, BIC-selected best-fit nuclear models, and SH-like aLRT branch support (-fast -st AA -m TEST -msub nuclear -alrt 1000). The resulting tree files were transformed into a format compatible with Phylopypruner v1.2.4, as detailed by Thalen et al. (https://pypi.org/project/phylopypruner/), using the assistance of ApplyPPPFilter (see key resources table – Sotware and Algorithms).

Orthologue sets were pruned using Phylopypruner v1.2.4 (Thalen et al., https://pypi.org/project/phylopypruner/) to remove paralogs (--mask pdist --prune MI --min-taxa 10 --trim-lb 5 --min-support 0.75 --min-gene-occupancy 0.1 --min-otu-occupancy 0.1 --threads 80 --trim-freq-paralogs 4 --trim-divergent 1.25 --min-pdist 1e-8 –jackknife), resulting in a set of 5,290 orthologues.

After applying the taxonomic filter using FilterPPPResult (-t 3) (see key resources table – Sotware and Algorithms), we identified and selected 2,258 loci. These loci underwent masking with PREQUAL^109^ v1.02. Following this step, we aligned them using MAFFT^105^ ginsi v7.304b with the utilization of a variable scoring matrix ('--allowshift --unalignlevel 0.8'), and any columns containing over 75% gaps were subsequently eliminated using ClipKIT^103^ v2.0.1.

The resultant trimmed alignments were then combined into a matrix consisting of 62 taxa and 420 loci.

To increase the number of informative loci, we repeated several steps and performed a second round of orthogroup sampling by using OSG with altered settings on the same set of orthogroups. For this, we changed the taxonomic filter applied to OSG to include 21 out of 40 taxa (38 ingroup, 2 outgroup) — with the aim of obtaining more informative sites from the ingroup. These were then filtered again as described above, in brief: PREQUAL, aligning with MAFFT, PhyloPyPruner, another taxonomic filtering with 2 out of 4 taxonomic groups ([i] chlorophytes, [ii] non-klebsormidiophycean streptophytes, [iii] *Klebsormidium* spp., and [iv] other Klebsormidiophyceae). We combined the resulting loci with the other 420 loci data using the Combine Orthogroup Sets tool (COGS, see GitHub). After another round of aligning with MAFFT, IQ-TREE, PhyloPyPruner, PREQUAL, applying MAFFT G-INS-I on the loci, IQ-TREE, and ClipKIT on the individual loci files, we concatenated the alignments with Phyx^121^ and applied ClipKIT to remove any columns that contained over 65% gaps. This yielded the final set of 845 loci. This matrix consisted of 90,321 aligned amino acid positions.

We inferred a maximum likelihood phylogeny with IQ-Tree2,^120^ multicore version 2.2.2.7. Best-fit nuclear models were selected based on the Bayesian Information Criterion (BIC). The tree was then reconstructed under the LG+C60 mixture model and 1000 replicates of both SH-like aLRT and ultrafast bootstrap approximation^122^ (UFBoot2) (-m LG+C60+G -s concatenated.fas -bb 1000 -alrt 1000).

#### Ancestral character state reconstruction

We used Phytools,^107^ which implements Yang's re-rooting method^110^ to perform ancestral character state reconstruction (ACSR) analyses about growth type and considered different character coding schemes, as well as symmetric and asymmetric character exchange rates, to examine the impact on the inferred ancestral character states. A first analysis utilized a 2-character state model, distinguishing between (1) unicellular and (2) multicellular *sensu lato* (including filamentous or multicellular forms), assuming ether symmetric (1:1) or asymmetric (2:1) rates between states. A second set of analysis employed a 4-character state model, differentiating between (1) unicellular, (2) coccoid, (3) filamentous, and (4) multicellular *sensu stricto*. We performed this either with symmetric and asymmetric rates. In all models, we assumed unordered states. We also performed ACSR of the habitat type as being either (1) terrestrial or (2) aquatic, and as being (1) humid or (2) arid, using a symmetric rates models.

#### Molecular clock

Bayesian molecular dating was performed with MCMCTree^123^ within the PAML^124^ package v4.9h. We used nine fossil calibrations with uniform prior distributions: The split between Chlorophytes and Streptophytes, Streptophyte crown group and five calibrations within land plants following parameterizations in Morris et al.^36^ (their Supplementary Table 8). Two additional nodes within streptophyte algae were calibrated; the ancestor of *Chara* and *Nitella* based on *Palaeonitella*, which has been interpreted as member of Characeae based on the presence of nodal discs and rhizoids^125^^,^^126^ as well as the ancestor of all Zygnematophyceae except *Spirogloea* based on *Rhyniotaenium velatum*, a saccoderm algae similar to *Mesotaenium* and *Serritaenia*.^127^ Both fossils derive from the Rhynie Chert formation, and thus calibrations were imposed at 407-480 Ma. We used the maximum likelihood tree topology (Figure 2) and removed *Isoetes* due to its unlikely recovered position in the outgroup. CorrTest^128^ did not reject the independent rates model on the maximum likelihood tree (score = 0.00024018; p > 0.05). Thus, we assumed a relaxed uncorrelated lognormal molecular clock model (clock = 2) and birth-death tree priors. Analyses used approximate likelihood calculations^129^ on the phylotranscriptomic dataset (single partition) under the LG+Γ model. A diffuse gamma Dirichlet prior was used for the prior on mean rates as 0.4444 replacements site^-1^ 10^8^ Myr^-1^ (‘rgene_gamma’: α = 2, β = 4.5). The rate drift parameter reflected considerable rate heterogeneity across lineages (‘sigma2_gamma’: α = 2, β = 2). A 100 Ma time unit was assumed. A preliminary MCMC analysis was run on the priors only (without sequence data) to ensure effective priors reflected biologically realistic constraints on calibrated nodes, which were largely overlapping (Figure S2B). The final analysis was run with two independent MCMC chains, each consisting of 100.02 million cycles, sampling every 5,000^th^ cycle after the first 20,000 cycles were excluded as burnin. Convergence was checked using Tracer^130^ v1.7.1; all parameters obtained effective sample sizeample sizes (ESS) > 200.

### Quantification and statistical analysis

The final phylogeny (Figure 2) was inferred under the LG+C60 model and a Shimodaira–Hasegawa-like approximate likelihood ratio test^131^ (SH-aLRT) with 1000 replicates as well as 1000 UFBOOT replicates. Additional phylogenomic analyses were performed by applying PMSF^108^ (part of the GitHub repository) mixture models that account for site-specific frequency variation. For the PMSF-based analyses, we used two guide trees inferred using the models LG+C60+F+Γ and LG+F+I+G4, in both cases using an SH-aLRT with 1000 replicates as well as 1000 UFBOOT replicates; these analyses yielded the same tree topology as the initial LG+C60.

As a stringent test of monophyly, we calculated gene jackknife^34^^,^^35^ proportions. Compared with pseudo-replicates from the non-parametric bootstrapping, gene jackknife pseudo-replicates represent more independent data subsets (gene alignments are resampled without replacement). This analysis allowed us to test the robustness of our preferred maximum likelihood tree with increased stringency, but also assess the effect of data subsampling, and the statistical support of individual branches to alignment length). We resampled gene alignments without replacement up to established lengths of 10,000, 30,000, 60,000 and 85,000 aligned amino acids. For each length, 100 pseudo-replicates were generated and analyzed by maximum likelihood (IQ-Tree under BIC-selected LG+Γ models) and the proportion of branches recovered by the gene jackknife samples were recorded.

We performed topology tests as implemented in IQ-TREE with 10,000 replicates of using the RELL method under the BIC-selected LG+Γ model. The following five topologies were tested: (i) unconstrained maximum likelihood tree (Figure 2), (ii) ((Hormidiellales, Entransiales), (*Streptofilum*, Entransiales, *Streptofilum*, (Klebsormidiales, Hormidiellales)), (iv) (Hormidiellales, *Streptofilum*, (Klebsormidiales, Entransiales)), (v) (*Streptofilum*, (Hormidiellales, (Klebsormidiales, Entransiales))). Alternative tree topologies were constructed by modifying the maximum likelihood tree and in the case of the multifurcation in iv, this was resolved by performing a constrained maximum likelihood search with the final alignment and best-fit model (LG+C60+Γ).

ModelFinder^106^ recovered that the LG+C60 mixture model was a better fitting model for protein evolution than the best-fitting standard model LG+F+I+G4 [LG+F+I+G4 Log(Likelihood) = -1551643.771; Bayesian Information Criterion of 3104907.921; LG+C60 Log(Likelihood) = -1542486.195; Bayesian Information Criterion of 3086364.547].

## Supplemental information

Document S1. Figures S1 and S2

Data S1. Ancestral character state reconstruction of body plan across more than 800 million years of klebsormidiophycean evolution and the phenylalanine ammonia-lyase gene family history, related to Figure 3 (A) To examine the ancestral character states of growth types in unicellular or multicellular organisms, coding schemes represented varying levels of complexity and hypotheses regarding the homology of growth types. The shown color-coded character state distributions represent yellow for unicellular growth, orange for sarcinoid growth, and purple for filamentous growth as well as multicellular growth sensu stricto; results of symmetric and asymmetric models are shown. (B) To examine the ancestral habitats of the Klebsormiodphyceae, we coded the habitat occurrence of the species as light blue for humid and white for arid. Note the arid-dwelling ancestor of the G-clade of *Klebsormidium* spp. (C) Same as Figure 3 but with an asymmetrical model for character changes. (D) Phylogenetic analysis of PAL with an expanded sampling in Klebsormidiophyceae. Sequences were sampled based on de Vries et al.,^59^ with a phylodiverse distribution of PAL and histidine ammonia lyase (HAL) sequences from (1) the green lineage (land plants, streptophyte algae and chlorophytes), (2) non-Chloroplastidial eukaryotes, and (3) bacteria. Based on these 364 PAL and HAL sequences in total, an alignment was computed using MAFFT v7.453 with a L-INS-I approach. IQ-TREE multicore version 1.5.5 was used to compute a maximum likelihood phylogeny with LG+F+G4, which was chosen according to Bayesian Information Criterion as best-fit-model using ModelFinder. 5,000 ultrafast bootstrap replicates were computed (shown in differently sized dots, see KEY)

Document S2. Article plus supplemental information
