# Supplementary material for: Phylogenomic insights into the first multicellular streptophyte
Source: Curr Biol. 2024 Feb 5;34(3):670–681.e7. doi: 10.1016/j.cub.2023.12.070 (PMC10849092; doi:10.1016/j.cub.2023.12.070)
Supplement: Document S1. Figures S1 and S2 [file mmc1.pdf]

**Current Biology, Volume *34***

## Supplemental Information

# Phylogenomic insights

# into the first multicellular streptophyte

**Maaike J. Bierenbroodspot, Tatyana Darienko, Sophie de Vries, Janine M.R. Fürst-Jansen, Henrik Buschmann, Thomas Pröschold, Iker Irisarri, and Jan de Vries**

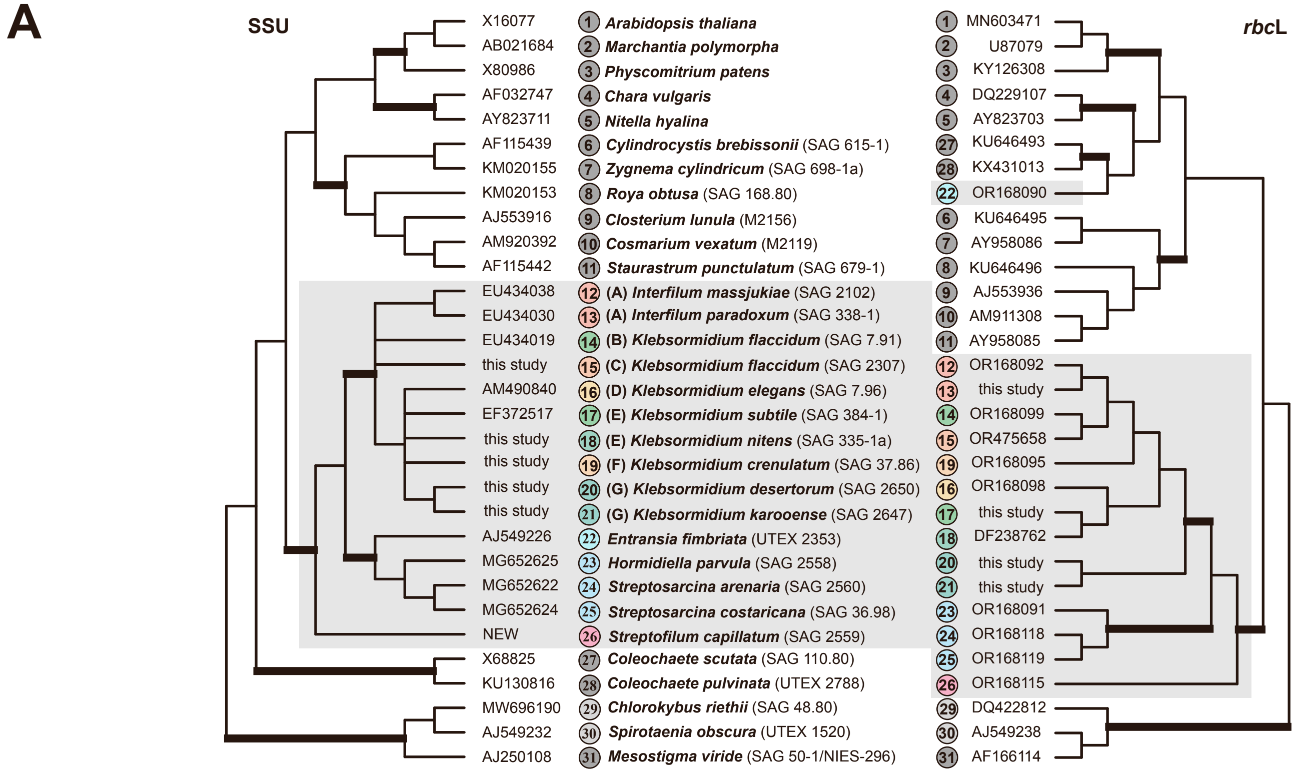


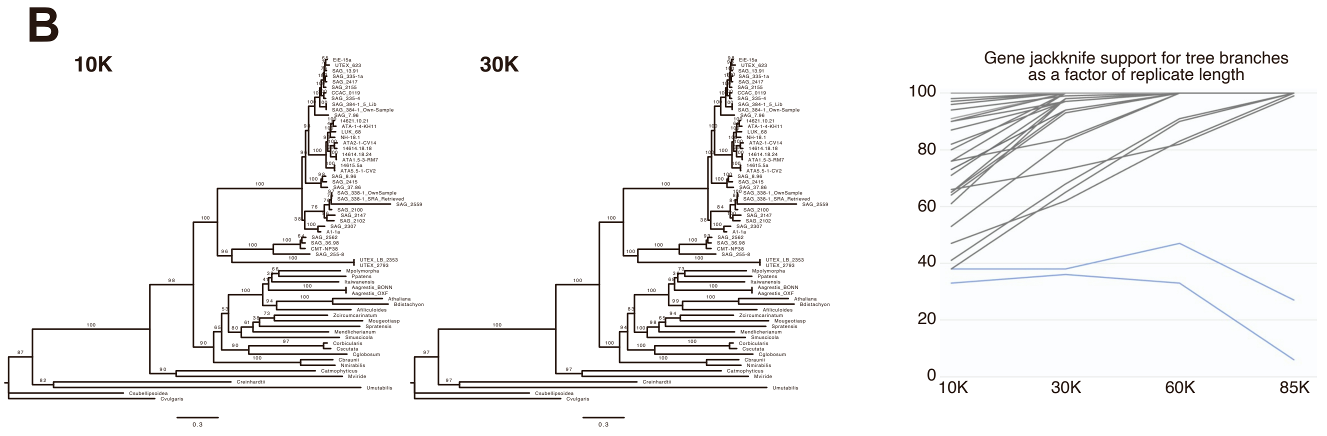


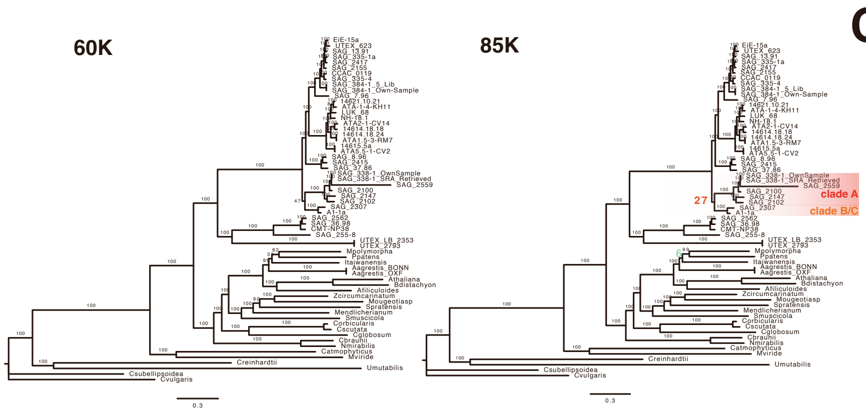


C

| Tree | logL | p-KH | p-SH | p-WKH | p-WSH | c-ELW | p-AU |
|---|---|---|---|---|---|---|---|
| Unconstrained tree | -1554434.6 | 0.21 | 0.843 | 0.21 | 0.21 | 0.5 | 0.211 |
| ((H,E)(S,K)) | -1557070.6 | 0 | 0 | 0 | 0 | 0 | 9.20E-06 |
| (E,(S,(K,H))) | -1559832.4 | 0 | 0 | 0 | 0 | 0 | 1.52E-34 |
| (H,(S,(K,E))) | -1559220.8 | 0 | 0 | 0 | 0 | 0 | 3.33E-05 |
| (S,(H,(K,E))) | -1559917.8 | 0 | 0 | 0 | 0 | 0 | 2.76E-06 |

**Figure S1: The topology of the streptophyte phylogeny with a focus on Klebsormidiophyceae, related to Figure 2. (A) Molecular phylogeny of the Streptophyta based on SSU rDNA and *rbc*L sequence comparisons.** The phylogenetic trees shown were inferred using the maximum likelihood method based on the data sets (31 taxa: 1794 aligned positions for SSU, 1428 for *rbc*L) using PAUP 4.0a169 (Swofford 2002). For the analyses the best model was calculated by the automated model selection tool implemented in PAUP. The setting of the best model was given as follows: (SSU) TrN+I+G (base frequencies: A 0.2453, C 0.2156, G 0.2823, T 0.2569; rate matrix A-C 1, A-G 2.0336, A-U 1, C-G 1, C-U 6.2892, G-U 1) with the proportion of invariable sites (I = 0.4778) and gamma shape parameter (G = 0.5542); (*rbc*L) GTR+I+G (base frequencies: A 0.2913, C 0.1835, G 0.1859, T 0.3392; rate matrix A-C 1.1839, A-G 6.1340, A-U 3.4723, C-G 1.2456, C-U 9.5079, G-U 1.0000) with the proportion of invariable sites (I = 0.5316) and gamma shape parameter (G = 2.222). The branches in bold are highly supported in all analyses (bootstrap values > 70% calculated with PAUP using maximum likelihood, neighbor-joining using maximum likelihoodsettings, and maximum parsimony). The classes are marked in the same colors as in the main Figure 2. **(B) Jackknife analysis.** Branch support measured by proportion of bipartitions recovered by gene jackknife pseudo-replicates (random sampling of gene alignments, without replacement) of increased sizes (1,000-85,000 aligned amino acids). **(C) Tests for alternative tree topologies.** The first column shows the seven tested topology tests (E = Entransiales, H = Hormidiellales, K = Klebsormidiales, S =Streptofilum), followed by log-likelihood values (logL) and probabilities for one sided Kishino-Hasegawa (KH), Shimodaira-Hasegawa (SH), weighted KH (WKH) and SH (WKH), the expected likelihood weight (ELW), and probability of the approximately unbiased (AU) test.

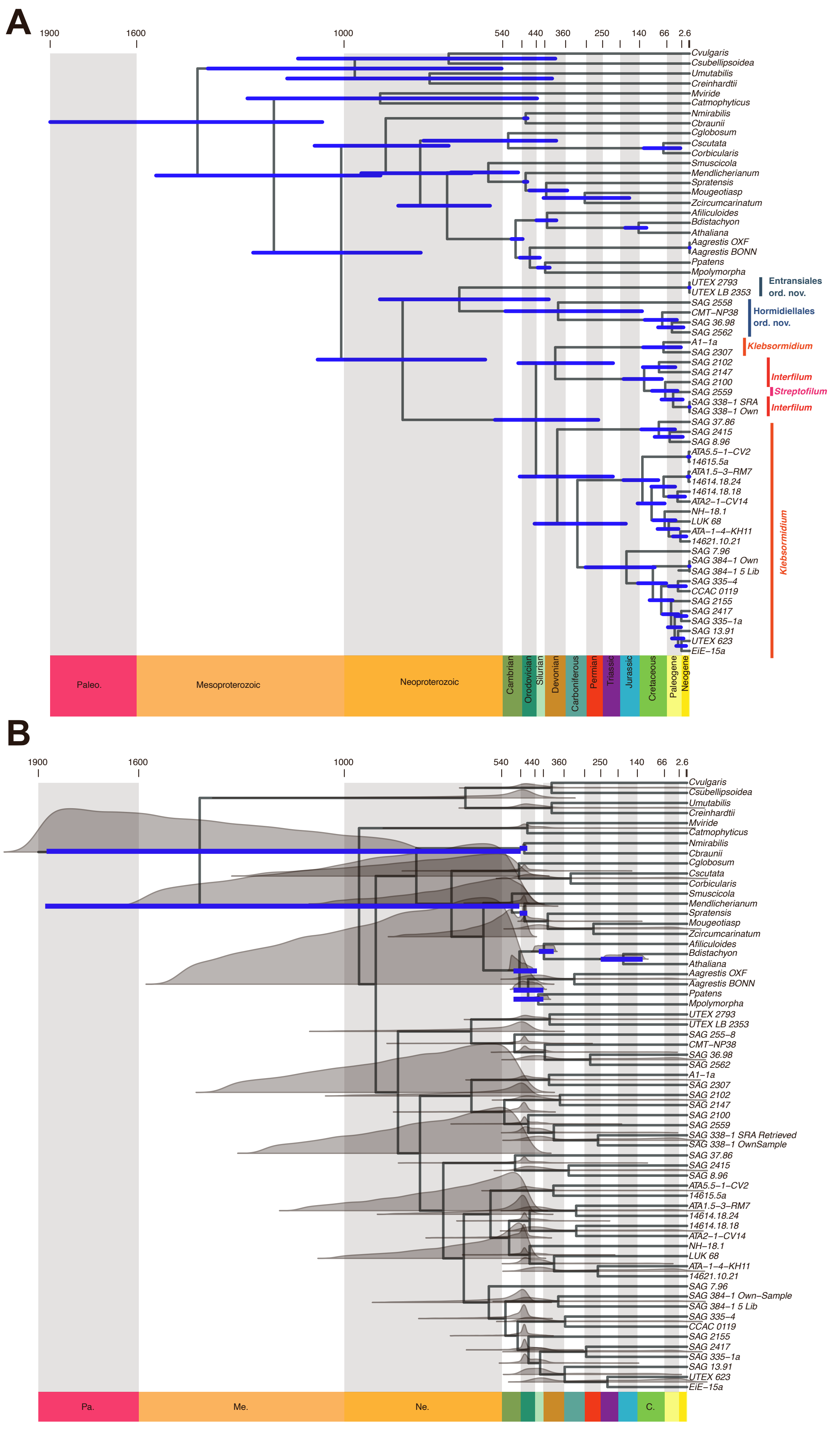


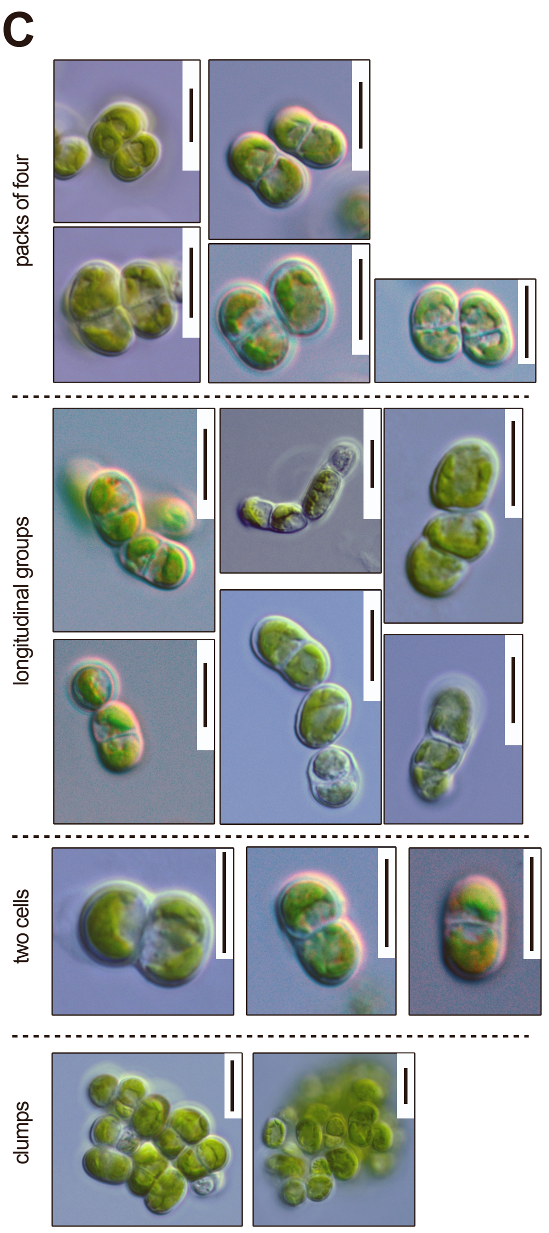


**Figure S2: (A) Molecular clock analysis of the Klebsormidiophyceae, related to Figures 2 and 3.** Estimates of divergence times in million years were calculated using Time Tree. A uniform distribution was applied. Chlorophytes served as outgroup. **(B) Effective priors.** Overlap between the calibrations (blue bars) and the effective prior density. **(C) Different morphologies of *interfilum*, related to Figure 3.** *Interfilum* SAG 2147 was grown on agar and diverse morphologies were observed using light microscopy. Scale bar = 10 $\mu$m in all pictures.
