# Supplementary figures and images for: Phylogenomic insights into the first multicellular streptophyte
Source: Curr Biol. 2024 Feb 5;34(3):670–681.e7. doi: 10.1016/j.cub.2023.12.070 (PMC10849092; doi:10.1016/j.cub.2023.12.070)

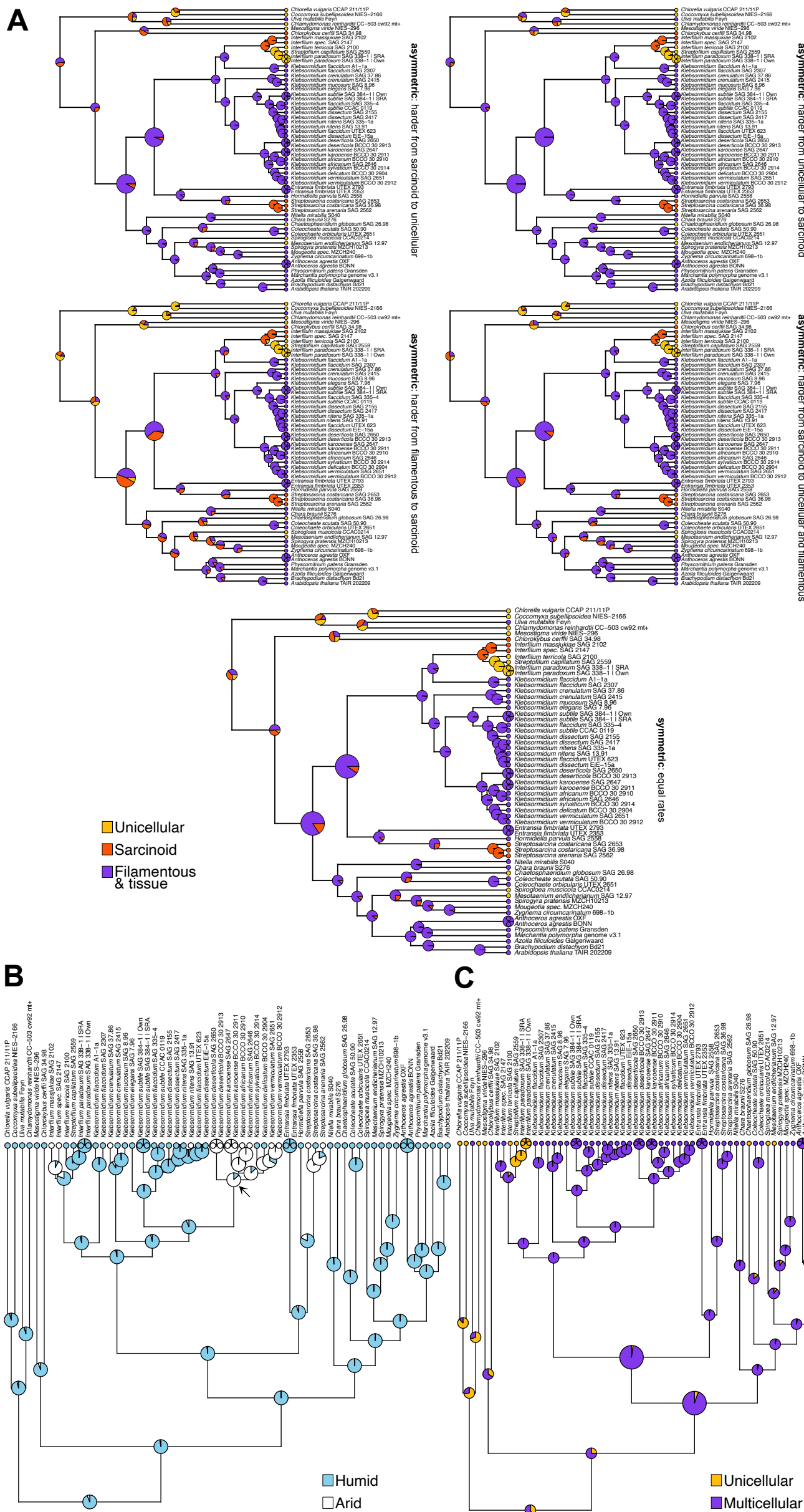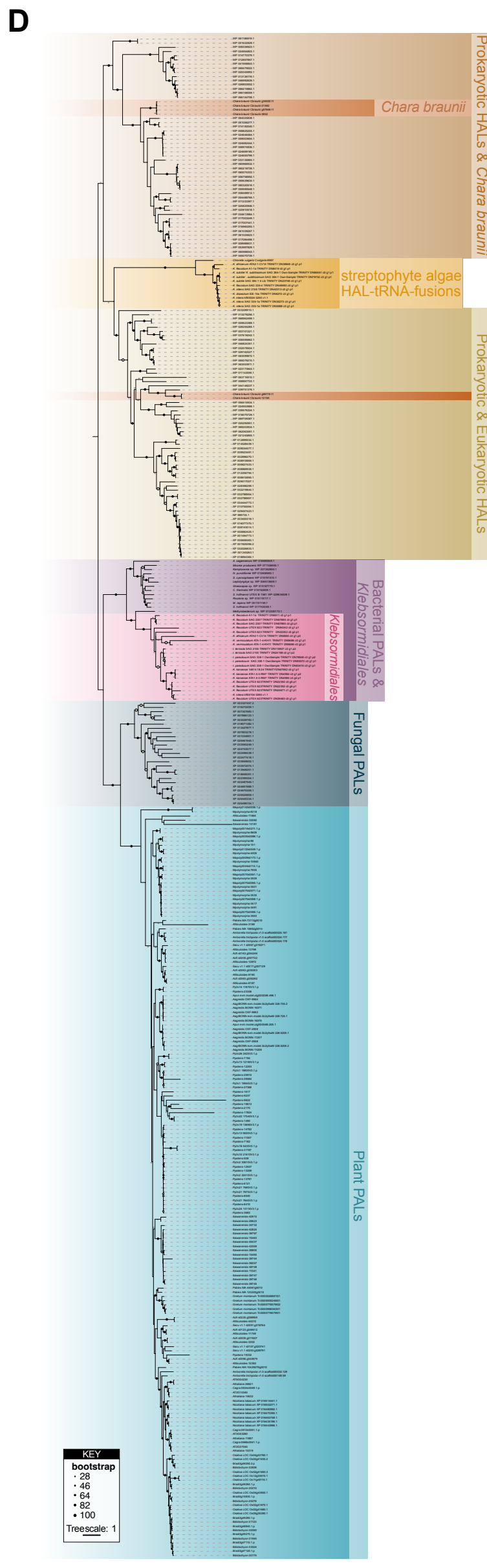

Supplement: Data S1. Ancestral character state reconstruction of body plan across more than 800 million years of klebsormidiophycean evolution and the phenylalanine ammonia-lyase gene family history, related to Figure 3 — (A) To examine the ancestral character states of growth types in unicellular or multicellular organisms, coding schemes represented varying levels of complexity and hypotheses regarding the homology of growth types. The shown color-coded character state distributions represent yellow for unicellular growth, orange for sarcinoid growth, and purple for filamentous growth as well as multicellular growth sensu stricto; results of symmetric and asymmetric models are shown. (B) To examine the ancestral habitats of the Klebsormiodphyceae, we coded the habitat occurrence of the species as light blue for humid and white for arid. Note the arid-dwelling ancestor of the G-clade of Klebsormidium spp. (C) Same as Figure 3 but with an asymmetrical model for character changes. (D) Phylogenetic analysis of PAL with an expanded sampling in Klebsormidiophyceae. Sequences were sampled based on de Vries et al.,59 with a phylodiverse distribution of PAL and histidine ammonia lyase (HAL) sequences from (1) the green lineage (land plants, streptophyte algae and chlorophytes), (2) non-Chloroplastidial eukaryotes, and (3) bacteria. Based on these 364 PAL and HAL sequences in total, an alignment was computed using MAFFT v7.453 with a L-INS-I approach. IQ-TREE multicore version 1.5.5 was used to compute a maximum likelihood phylogeny with LG+F+G4, which was chosen according to Bayesian Information Criterion as best-fit-model using ModelFinder. 5,000 ultrafast bootstrap replicates were computed (shown in differently sized dots, see KEY) [file mmc2.pdf]
